# Simplified Anaerobic Cultivation of *Acetivibrio cellulolyticus* and *Methanosarcina barkeri*: Implications for Lignocellulosic Biomethane Research

**DOI:** 10.3390/bioengineering13090960

**Published:** 2026-08-23

**Authors:** Vaibhavi Bele, Adrien Rizzi, Debra M. Hausladen, Inès Esma Achouri

**Affiliations:** 1 Group of Research on Technologies and Processes (GRTP), Department of Chemical and Biotechnological Engineering, Université de Sherbrooke, Sherbrooke, QC J1N 0J8, Canada; 2 CRB Innovations Inc., Westbury, QC J0B 1R0, Canada; 3 Department of Civil and Building Engineering, Université de Sherbrooke, Sherbrooke, QC J1N 0J8, Canada

**Keywords:** anaerobes, methane, lignocellulose, biomass, microcrystalline cellulose, anaerobic cultivation, archaea, gas production, electron microscopy

## Abstract

Conventional anaerobic digestion relies on diverse inocula present in sludge-based systems. A defined consortium approach was investigated as an alternative. *Acetivibrio cellulolyticus* was chosen as the cellulose degrader, and two strains of *Methanosarcina barkeri* were selected as methane producers. Initial cultivation following manufacturer protocols highlighted significant challenges in maintaining strict anaerobic conditions, particularly in the absence of specialized infrastructure. A simplified anaerobic cultivation workflow was therefore evaluated for pure cultures of the selected anaerobes and subsequently used to evaluate a defined consortium using microcrystalline cellulose (MCC) and industrial lignocellulosic biomass (LB) residue as growth substrates. The workflow enabled successful cultivation of pure cultures in their recommended nutrient media without detectable contamination, as assessed by microscopy, aerobic contamination checks, and gas chromatography analysis. Growth-associated observations were obtained on MCC after prolonged incubation (~30 days); however, a metabolically active cellulolytic–methanogenic consortium was not established, as methane was not detected and no activity was detected on the LB substrate. This study demonstrates that anaerobic cultivation of fastidious microorganisms is feasible using a simplified method without fully controlled anaerobic environments and highlights inherent challenges associated with the defined consortium on substrates such as MCC and complex LB residue. The simplified workflow may provide an accessible approach to anaerobic cultivation for sustainable biomethane research in laboratories lacking specialized anaerobic infrastructure. Further work is required to determine conditions supporting methane production using the defined consortium.

## 1. Introduction

Lignocellulosic biomass (LB), derived from forest residues, is a renewable feedstock of interest for sustainable bioenergy production. However, its conversion is challenging because of the recalcitrant nature of lignin and the high crystallinity of cellulose. Industrial processing commonly involves dilute-acid steam explosion followed by lignin extraction, generating a cellulose-rich residue that can serve as a substrate for bioenergy production [1]. Anaerobic digestion (AD) is widely used to convert organic substrates into biogas, primarily methane (CH_4_) and carbon dioxide (CO_2_), through the activity of complex microbial communities. Conventional AD systems rely on microbial consortia present in sludge, whose metabolic diversity contributes to process robustness and industrial applicability. However, the complexity of these communities limits precise control of microbial interactions and metabolic pathways. A proposed alternative is the use of defined microbial consortia composed of a small number of selected anaerobic microorganisms. Such systems may provide greater control over microbial interactions and facilitate mechanistic studies of cellulose-to-methane conversion while potentially reducing reliance on sludge-based systems.

Previous studies have demonstrated the feasibility of defined anaerobic consortia for methane production from cellulose. For example, a four-species consortium consisting of *Ruminiclostridium cellulolyticum* (cellulolytic bacteria), *Methanospirillum hungatei*, *Methanosaeta concilii*, and *Desulfovibrio vulgaris* (sulphate-reducing bacteria) produced more methane than simpler community configurations through metabolic cross-feeding on cellulose [2]. Similarly, *Acetivibrio cellulolyticus* degrades cellulose to acetate, ethanol, H_2_, and CO_2_, while co-cultivation with *Methanosarcina barkeri* promotes methane production through syntrophic interactions [3]. In the presence of H_2_, CO_2_, and acetate, *M. barkeri* preferentially utilizes gaseous substrates, with acetate becoming a primary substrate only after substantial gas consumption. Hydrogen partial pressure was also found to influence ethanol and acetate metabolism [3]. Together, these studies demonstrate that pairing cellulolytic bacteria with methanogenic archaea can support cellulose-to-methane conversion [2,3].

**Figure 1:**
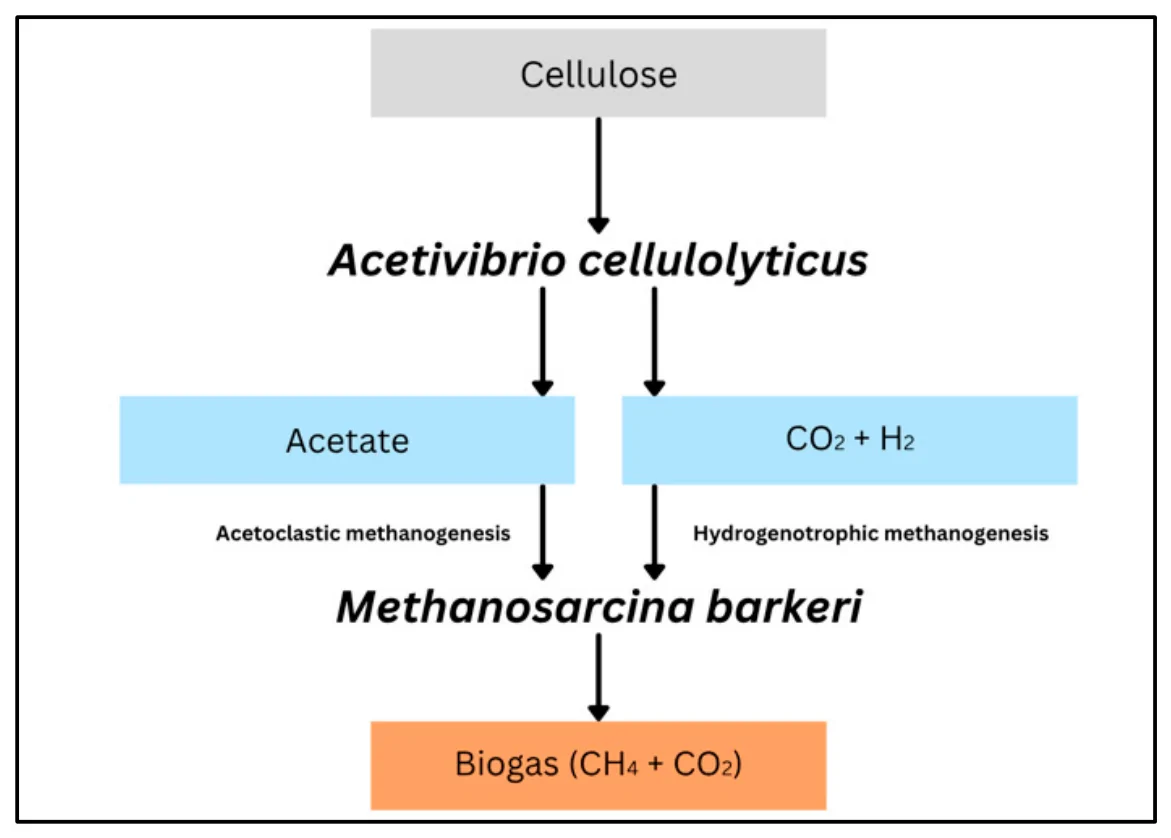
Simplified depiction of the microbial relationship between *A. cellulolyticus* and *M. barkeri* involved in biogas production from cellulose; *A. cellulolyticus* degrades cellulose and produces acetate, H_2_, and CO_2_. *M. barkeri* converts these intermediates to methane through acetoclastic and hydrogenotrophic methanogenesis, resulting in biogas production.

The ability of *A. cellulolyticus* to degrade crystalline cellulose, including Avicel, Whatman CF-11, and microcrystalline cellulose (MCC), is well documented [4], making it a suitable cellulolytic member for a defined consortium. *M. barkeri* was selected because it is metabolically versatile and capable of acetoclastic, hydrogenotrophic, and methylotrophic methanogenesis [5,6,7]. Among these pathways, hydrogenotrophic methanogenesis is the most thermodynamically favorable (Table 1). This metabolic flexibility enables *M. barkeri* to utilize multiple intermediates generated during cellulose degradation, including acetate, H_2_, and CO_2_. Previous co-culture studies have demonstrated syntrophic interactions between *A. cellulolyticus* and *M. barkeri* [3,8], providing the rationale for selecting these organisms for the present study. The expected metabolic interactions between the cellulolytic bacterium and methanogen are illustrated in Figure 1.

Despite these advances, the performance of defined consortia remains highly dependent on cultivation conditions and substrate characteristics, particularly when transitioning from model substrates such as MCC to complex lignocellulosic residues. A critical but often overlooked challenge is the cultivation and maintenance of the strictly anaerobic microorganisms required for such systems. Even brief oxygen exposure can adversely affect many anaerobes, necessitating specialized equipment, anaerobic workspaces, and carefully controlled handling procedures. Although numerous anaerobic cultivation techniques have been developed since the introduction of the Hungate method [9], cultivation of fastidious anaerobes remains technically demanding and can be challenging for laboratories lacking dedicated anaerobic infrastructure. Several approaches have been proposed to simplify anaerobic media preparation and cultivation [9,10,11]; however, their applicability to cellulolytic and methanogenic microorganisms relevant to lignocellulosic biomethane production remains insufficiently explored.

In this study, a simplified anaerobic cultivation workflow was evaluated for the cultivation of pure cultures of *Acetivibrio cellulolyticus* and two strains of *Methanosarcina barkeri* without specialized anaerobic infrastructure. The cultivated microorganisms were subsequently evaluated as members of a defined cellulolytic–methanogenic consortium using MCC as a model substrate and an industrial LB residue as a complex lignocellulosic substrate. To the best of our knowledge, such a workflow has not previously been evaluated for these organisms in the context of lignocellulosic biomethane research. The study assessed both the feasibility of simplified anaerobic cultivation and the limitations associated with applying a defined consortium approach to complex lignocellulosic substrates.

## 2. Materials and Methods

### 2.1. Materials, Organisms, and Growth Media

Anaerobic cultivation was performed in 100- and 125 mL glass vials (MilliporeSigma Canada Ltd., Wheaton glass serum bottle, cat. no. Z114006 and cat. no. Z114014, respectively), ≈15 mL Hungate tubes (VWR International Co, cat. no. 89167-170, Supplier: Chemglass, Supplier Number: CLS-4208-01) and ≈30 mL Balch tubes (VWR International Co, cat. no. 89167-178, Supplier: Chemglass, Supplier Number: CLS-4209-01), closed with butyl rubber septa and sealed using 20 mm aluminum crimp seals. The anaerobic strains were obtained from the Leibniz Institute DSMZ-German Collection of Microorganisms and Cell Cultures GmbH and American Type Culture Collection (ATCC) (supplied by Cedarlane Laboratories Limited, the exclusive Canadian distributor for ATCC). *Methanosarcina barkeri* Schnellen ATCC 43569 was maintained in ATCC medium 2889 (SAB broth) and incubated at 37 ± 1 °C for 14 days. *Acetivibrio cellulolyticus* DSM 1870 was maintained in DSMZ medium 165 and incubated at 37 ± 1 °C for 8–14 days.

*Methanosarcina barkeri* Fusaro ATCC BAA-2329 was maintained in ATCC medium 2467 (MS-OCM base medium) using only the simplified cultivation method under an H_2_:CO_2_ 4:1 headspace at 20 psi gauge pressure (137.9 kPa). Cultures were incubated at 30 ± 1 °C for 10 days for the first three subcultures, and subsequent subcultures were incubated at 37 ± 1 °C.

Sterile vitamin and trace mineral solutions based on Wolfe’s formulation were obtained from ATCC (MD-VS^TM^ and MD-TMS^TM^).

### 2.2. Simplified Anaerobic Cultivation Method

**Figure 2:**
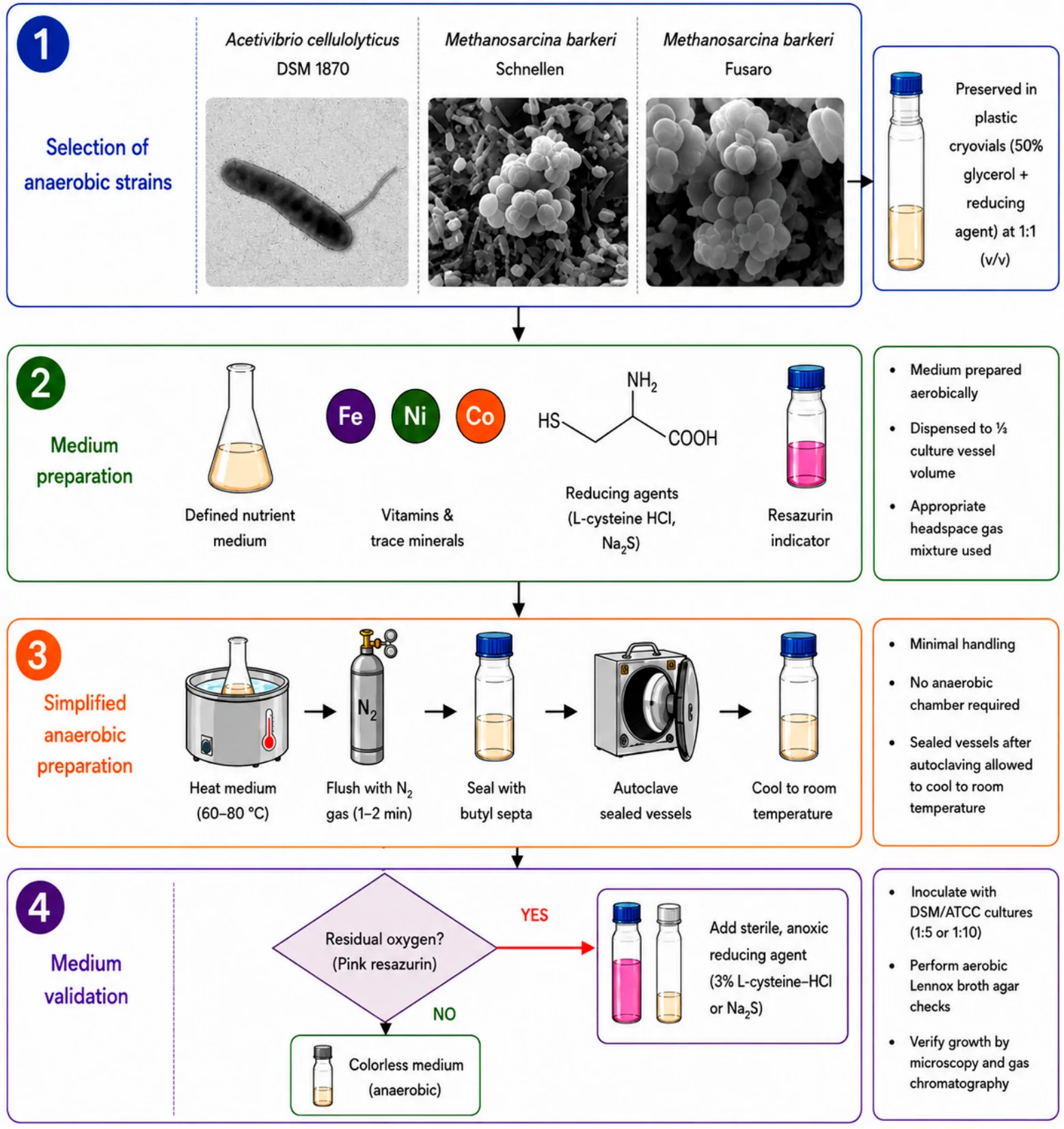
Simplified anaerobic cultivation workflow adopted in this study.

A simplified anaerobic cultivation workflow based on previously reported methods was adopted [5,10,11]. The components for the desired nutrient medium were mixed in a bottle under aerobic conditions, including the resazurin indicator dye (1 mg L^−1^), reducing agent, trace elements, vitamins, and mineral solutions, and the pH was adjusted. The medium was transferred to anaerobic vessels at no more than one-third of the vessel volume. Vessels were then inserted into a hot-water bath at 60–80 °C, flushed with N_2_ gas for 1–2 min, capped using butyl rubber stoppers, and sealed with 20 mm aluminum crimp seals. Subsequent preparations were performed using a 50 °C water bath. Lastly, cultivation vessels were autoclaved at 121 °C for 20 min using the liquid cycle. Figure 2 illustrates the simplified anaerobic cultivation workflow used in this study.

Sealed vessels were autoclaved using appropriate laboratory procedures with safety precautions for pressurized containers. If any vessel lost its seal during sterilization, it was discarded.

Resazurin is a redox indicator commonly used in anaerobic cultivation and serves as an indicator of residual oxygen in the medium by turning pink in the presence of oxygen. If necessary, an additional reducing agent (3% L-cysteine·HCl·H_2_O, 0.1 mL per 10 mL of medium) was added to the medium to remove residual oxygen. Cell viability and active morphology of the stock cultures were verified by light microscopy prior to inoculation. Before use, syringes were rendered anoxic by repeatedly drawing and expelling sterile reduced water from sealed vessels containing an N_2_ headspace, thereby displacing residual air within the syringe and needle.

All anaerobes used in this study were cryopreserved at −80 °C using a stock solution of 50% sterile anoxic glycerol (equilibrated in a hot-water bath and flushed with N_2_ gas) containing 3% L-cysteine·HCl·H_2_O as the reducing agent (0.1 mL per 10 mL of freezing medium). Plastic cryovials (Corning^TM^ 2 mL) were used to mix culture and glycerol solution at a 1:1 (*v*/*v*) ratio, resulting in a final glycerol concentration of 25% (*v*/*v*). Cryopreservation was performed without an anaerobic chamber.

### 2.3. Use of MCC and LB as C Sources for a Defined Consortium

Following the simplified cultivation workflow, active cultures were subcultured every four days for 2–3 weeks in their respective recommended nutrient media before experiments using MCC and LB residue. Four-day-old pre-cultures were used as inoculum. Cultures were inoculated at 2% (*v*/*v*) into the medium described below and incubated at 37 ± 1 °C for 21–28 days. All experiments were performed in triplicate biological replicates.

Industrial lignocellulosic biomass (LB) residue was used as a complex real-world C substrate, along with MCC (Avicel) as the model control. The industrial LB residue was obtained after dilute-acid steam explosion of wood chips followed by lignin extraction and used on a dry-weight basis. More details are reported elsewhere [12]. The defined consortium or co-culture consisted of either *M. barkeri* Fusaro + *A. cellulolyticus* or *M. barkeri* Schnellen + *A. cellulolyticus*. Blanks (uninoculated nutrient media only) and monocultures of *A. cellulolyticus* were used as controls. Monocultures of either *M. barkeri* strain were not included because methanogens cannot directly utilize MCC or LB residue as sole carbon substrates.

To evaluate growth on these carbon sources, the simplified cultivation workflow was used together with the following medium described by Khan et al. [13] (formulation IV) (for 1 L of medium): K_2_HPO_4_ 0.296 g, KH_2_PO_4_ 0.18 g, NH_4_Cl 0.68 g, (NH_4_)_2_SO_4_ 0.15 g, MgSO_4_·7H_2_O 0.09 g, CaCl_2_·2H_2_O 0.06 g, NaHCO_3_ 2.06 g, FeSO_4_·7H_2_O 0.02 g, FeCl_2_·4H_2_O 0.0656 g, trace mineral solution 10 mL, vitamin solution 10 mL, Na_2_S·9H_2_O 0.25 g, L-cysteine·HCl·H_2_O 0.25 g, resazurin 1 mg, MCC (Avicel) or LB 5 g, pH 6.7–7.0 with N_2_ sparging. Trace mineral solution contained (mg L^−1^): nitrilotriacetic acid, 1500; MgSO_4_·7H_2_O, 3000; MnSO_4_·H_2_O, 500; NaCl, 1000; FeSO_4_·7H_2_O, 100; CoCl_2_·6H_2_O, 100; CaCl_2_·2H_2_O, 100; ZnSO_4_·7H_2_O, 100; CuSO_4_·5H_2_O, 10; AlK(SO_4_)_2_·12H_2_O, 10; H_3_BO_3_, 10; and Na_2_MoO_4_·2H_2_O, 10. The vitamin solution contained (mg L^−1^): pyridoxine·HCl, 10; thiamine·HCl, 5; riboflavin, 5; nicotinic acid, 5; p-aminobenzoic acid, 5; biotin, 2; folic acid, 2; and vitamin B_12_, 0.5.

### 2.4. Analytical Methods

Gram staining was performed, and a light microscope (Leica DM1000, Richmond Hill, ON, Canada) was used with a 100× oil-immersion objective.

A benchtop scanning electron microscope (SEM) was used to study the morphology of the LB residue using a Phenom XL ThermoFisher G1 SEM (Waltham, MA, USA) with accelerating voltage ranging from 5 to 15 kV, equipped with detectors for secondary electrons (SE) and backscattered electrons (BSE). A transmission electron microscope (TEM) JEOL JEM-2100plus TEM (Peabody, MA, USA) at 80 kV was used. Sample preparation protocols are described in Appendix A.

Optical density measurements were not suitable because maintaining strict anaerobic conditions, together with precipitate formation and gas bubbles, interfered with reliable measurements.

Gas chromatography was therefore used to monitor microbial activity through headspace gas composition. The concentrations (mol %) of methane (CH_4_), hydrogen (H_2_), carbon dioxide (CO_2_), and nitrogen (N_2_) were measured using a gas chromatograph (456-GC Scion) equipped with two thermal conductivity detectors (TCDs) set at 150 °C. The front TCD was used to analyze CH_4_, H_2_, and N_2_, whereas the middle TCD was used for CO_2_ analysis. The reference N_2_ flow rate was 5 mL min^−1^ for both detectors. Hydrogen and argon were used as carrier gases for the front and middle TCDs, respectively. The injector and oven temperatures were set at 200 °C and 50 °C. Gas compositions were quantitatively interpreted only within the validated calibration ranges of the method (Appendix A.2). Measurements below the lower calibration limit were not interpreted and were reported as “not detected” (n.d.) where appropriate.

From actively growing cultures, 3 mL of headspace gas was sampled using a sterile needle and syringe after disinfecting the vessel septum with 70% ethanol. Data are presented as the average ± standard deviation of the three biological replicates.

## 3. Results

### 3.1. Limitations of Standard Protocols

**Figure 3:**
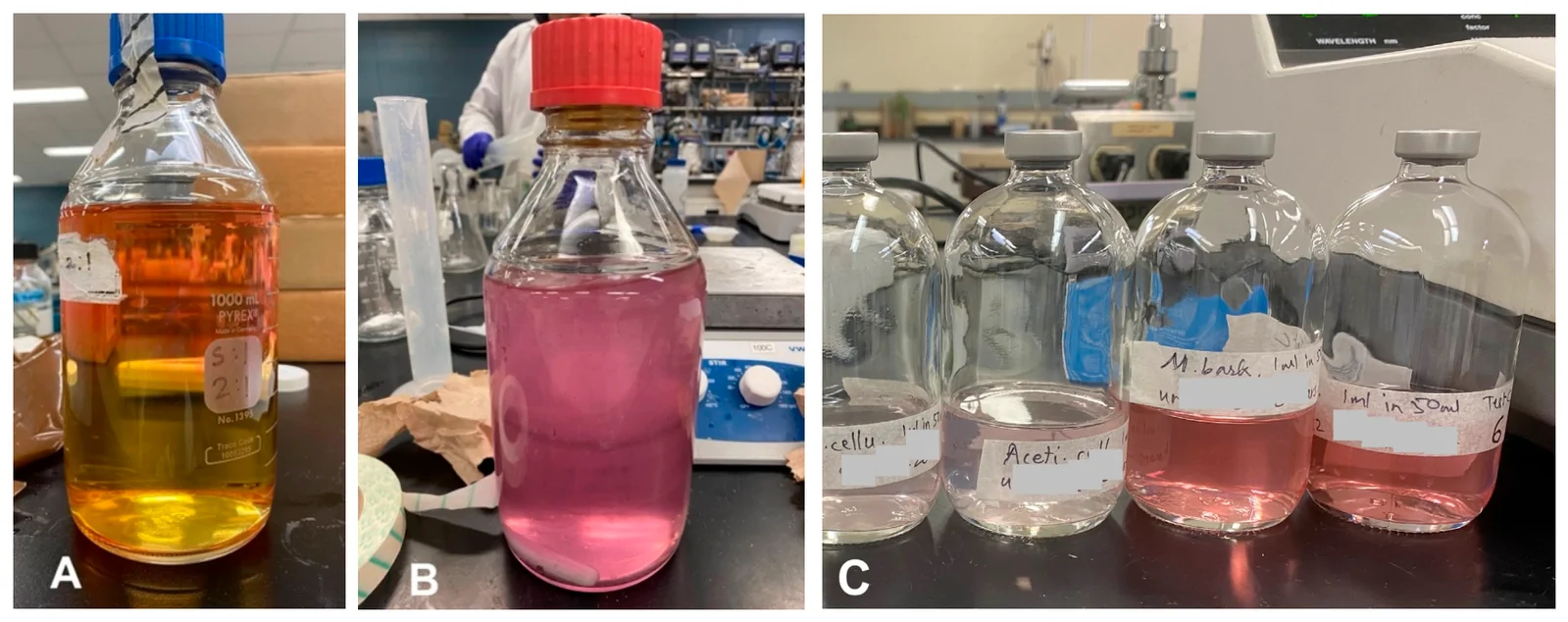
Media preparation using standard protocols. (**A**) Post-autoclave medium showing a pink/red zone near the headspace, indicating oxygen exposure. (**B**) Medium after 45–50 min of N_2_ sparging, with resazurin remaining pink, indicating incomplete reduction. (**C**) Post-autoclave medium in sealed glass serum vials exhibiting pink coloration after addition of filter-sterilized stock solutions, indicating residual oxygen in the medium.

Initial cultivation attempts were performed according to the manufacturer-recommended anaerobic cultivation procedures. Despite prolonged N_2_ sparging (45–60 min) and preparation of anaerobic stock solutions, evidence of residual oxygen was repeatedly observed in the media. Figure 3 shows representative examples of incomplete medium reduction. Following autoclaving, a red/pink zone was visible near the headspace (Figure 3A). Similarly, resazurin remained pink after 50 min of N_2_ sparging (Figure 3B), indicating incomplete reduction. In addition, media prepared using anaerobic filter-sterilized stock solutions occasionally developed pink coloration after sterilization (Figure 3C), suggesting oxygen intrusion during preparation.

**Figure 4:**
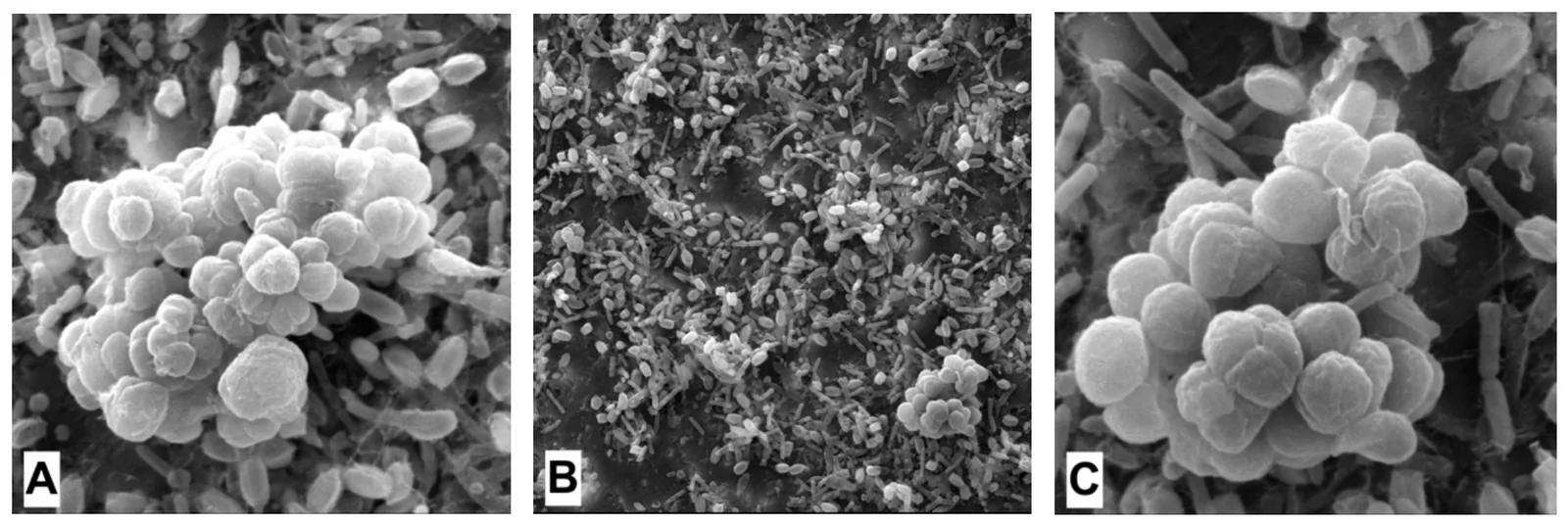
Electron microscopy images of a 15-day-old *Methanosarcina barkeri* Schnellen culture grown on the manufacturer-recommended medium. (**A**) Methanogen aggregates with surrounding microbial contamination, 5 µm scale. (**B**) Large methanogen aggregate associated with contaminating rod-shaped and coccoid microorganisms, 10 µm scale. (**C**) Higher-magnification view of the methanogen aggregate shown in (**B**), 3 µm scale.

Subsequent cultivation attempts resulted in viable cultures; however, contamination was observed in some preparations. Contamination likely occurred during the addition of filter-sterilized stock solutions to anaerobic, autoclave-sterilized media. Figure 4 shows electron microscopy images of a 15-day-old culture of *Methanosarcina barkeri* Schnellen, showing the characteristic formation of cloud-like aggregates, along with contamination by rod- and cocci-shaped microorganisms.

Together, these observations indicated that the standard cultivation workflow was susceptible to oxygen exposure and contamination, motivating the development of the simplified cultivation method.

### 3.2. Validation of Simplified Anaerobic Cultivation Workflow

**Figure 5:**
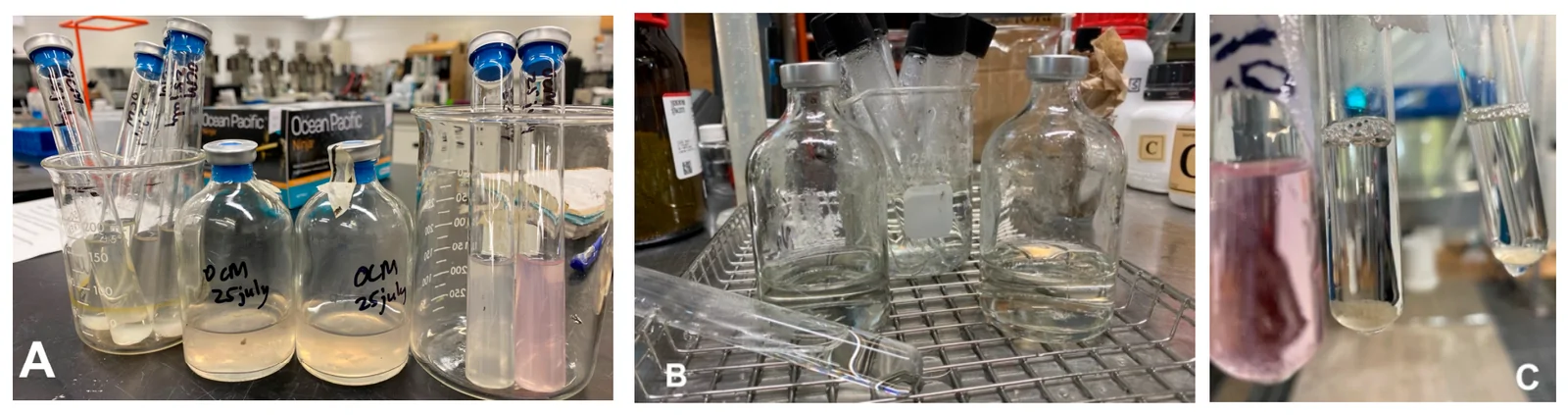
Validation of the simplified anaerobic cultivation workflow. (**A**) Anaerobic vessels following sterilization, showing residual oxygen in only one Balch tube as indicated by pink/purple resazurin coloration. (**B**) Completely reduced, colorless media in Hungate tubes and serum vials following medium preparation. (**C**) *Methanosarcina barkeri* Schnellen cultures in Hungate tubes; growth is visible as a biofilm deposit in reduced tubes, whereas the oxygen-contaminated tube (pink) showed no visible growth.

Following implementation of this workflow, anaerobic media were routinely obtained in a reduced state. Several independent cultivation attempts were performed over a one-year period in two different laboratories. Active cultures were successfully propagated through repeated transfers into freshly prepared nutrient media, demonstrating that the simplified cultivation workflow was reproducible for routine transfer of actively growing cultures. However, the number of successful and unsuccessful cultivation attempts was not systematically recorded. Figure 5 presents representative cultivation results obtained using the simplified workflow. Vessels showing incomplete reduction, indicated by pink or purple resazurin coloration (Figure 5A), were either supplemented with additional reducing agent (3% L-cysteine·HCl·H_2_O; 0.1 mL per 10 mL of medium) or discarded. Addition of the reducing agent restored complete medium reduction, resulting in colorless media (Figure 5B). Cultivation using the simplified workflow resulted in successful growth of all anaerobic strains without detectable contamination, as verified by microscopy, aerobic LB agar checks, and gas chromatography. Growth of *Methanosarcina barkeri* Schnellen was observed only in reduced media, whereas oxygen-contaminated tubes remained devoid of visible growth (Figure 5C). These observations confirmed that the simplified workflow was effective for establishing and maintaining pure anaerobic cultures.

### 3.3. Growth of Anaerobes

**Figure 6:**
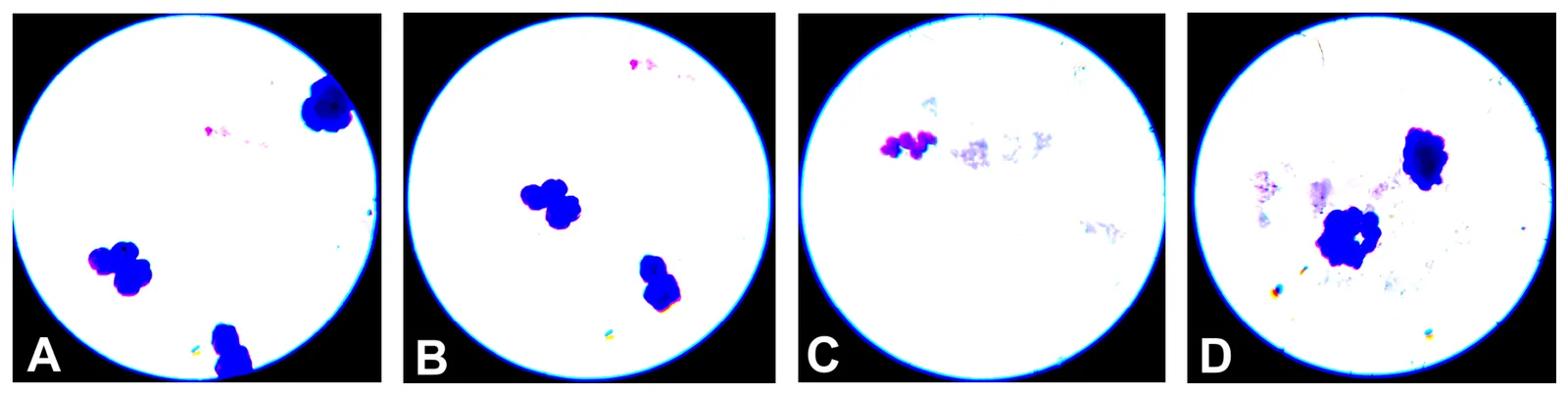
The two strains of methanogens as observed under a light microscope following crystal violet staining and a 100× oil-immersion objective: (**A**,**B**) day 9 culture of the Fusaro strain and (**C**,**D**) day 11 culture of the Schnellen strain. Images were adjusted for contrast and exposure.

Table 2 summarizes the headspace gas composition of pure anaerobic cultures after approximately 20 days of incubation in ~30 mL Balch tubes (10 mL working volume, 20 mL headspace). *A. cellulolyticus* produced higher concentrations of CO_2_ and H_2_ when grown on cellobiose than on MCC. Methane was detected in both *M. barkeri* strains, with *M. barkeri* Schnellen producing 50.26 ± 0.46 mol % CH_4_ and *M. barkeri* Fusaro producing 47.73 ± 0.40 mol % CH_4_ after 20 days. Residual CO_2_ and H_2_ remained in the headspace due to the gas mixture supplied during cultivation. Figure 6 shows microscopy images of the two strains of *M. barkeri* on their respective media with 4:1 H_2_:CO_2_ headspace gas: (Figure 6A,B) Fusaro strain and (Figure 6C,D) Schnellen strain; images edited for contrast and exposure.

**Figure 7:**
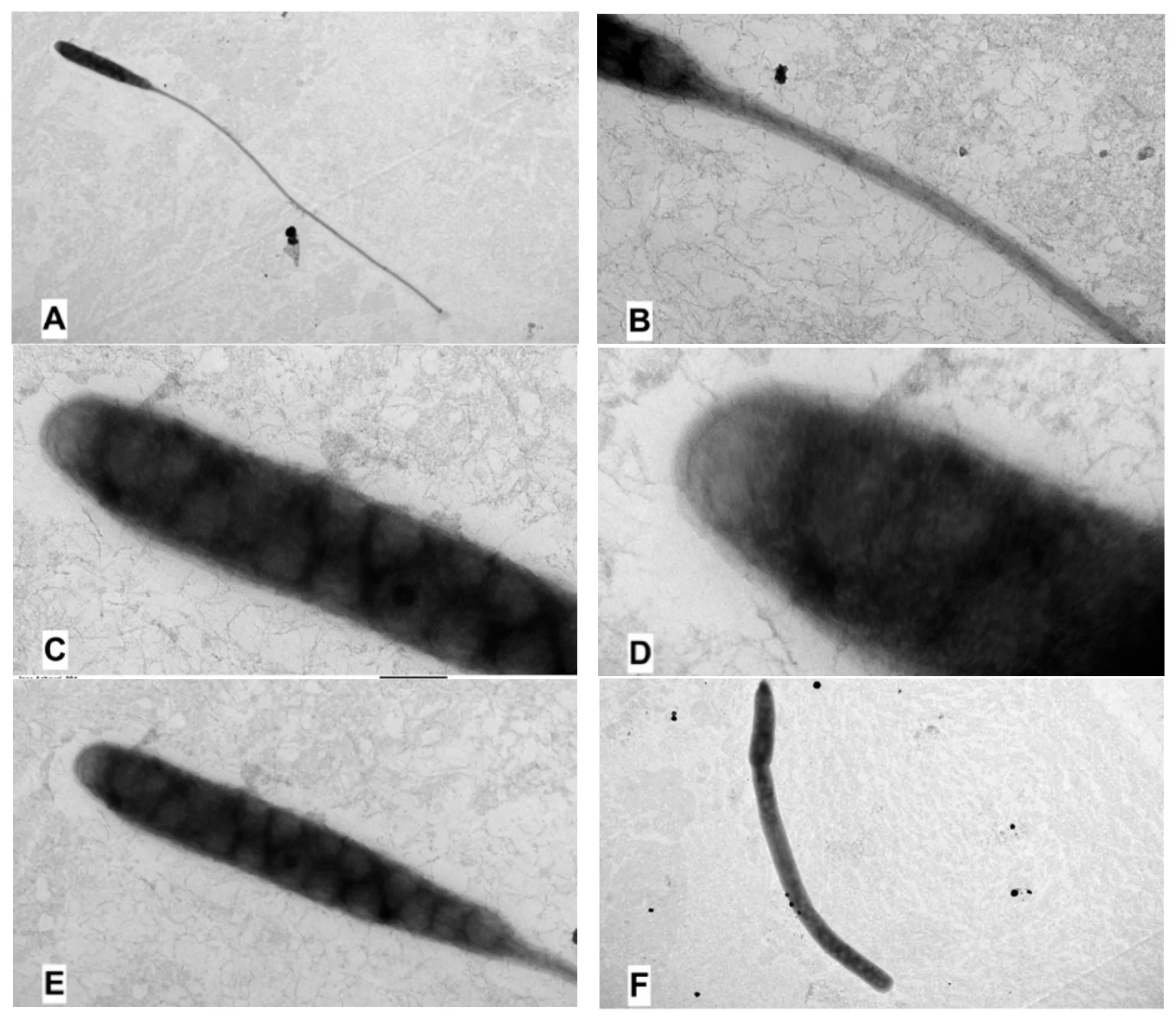
TEM images of *A. cellulolyticus* grown on cellobiose. (**A**) Rod-shaped cell with a long flagellum, 1 µm scale. (**B**) Flagellar attachment region, 200 nm scale. (**C**,**D**) Higher-magnification views of the cell body showing the cell-envelope structure, 200 nm and 100 nm scale, respectively. (**E**) Cell body excluding the flagellum, 200 nm scale. (**F**) Additional elongated cell with similar morphology, 2 µm scale.

*A. cellulolyticus* DSM 1870 cells appeared as straight to slightly curved rods and were occasionally observed with a flagellum. Following Gram staining, cells showed blue/purple coloration under light microscopy. Figure 7 presents TEM images at multiple scales (1 µm to 100 nm) of a two-week-old culture grown on cellobiose. A rod-shaped cell with a long flagellum was observed (Figure 7A–E). Higher-magnification images showed a visible cell-envelope structure along the cell body (Figure 7C,D). An additional elongated cell with similar morphology is shown in Figure 7F.

### 3.4. Growth on MCC and LB Substrate

Table 3 presents the headspace gas composition of 4-day-old pure cultures after repeated transfers in 100 mL glass vials (30 mL working volume, 70 mL headspace). *A. cellulolyticus* grown on cellobiose produced higher CO_2_ and H_2_ concentrations than cultures grown on MCC, whereas both *M. barkeri* strains produced methane under their respective growth conditions.

**Figure 8:**
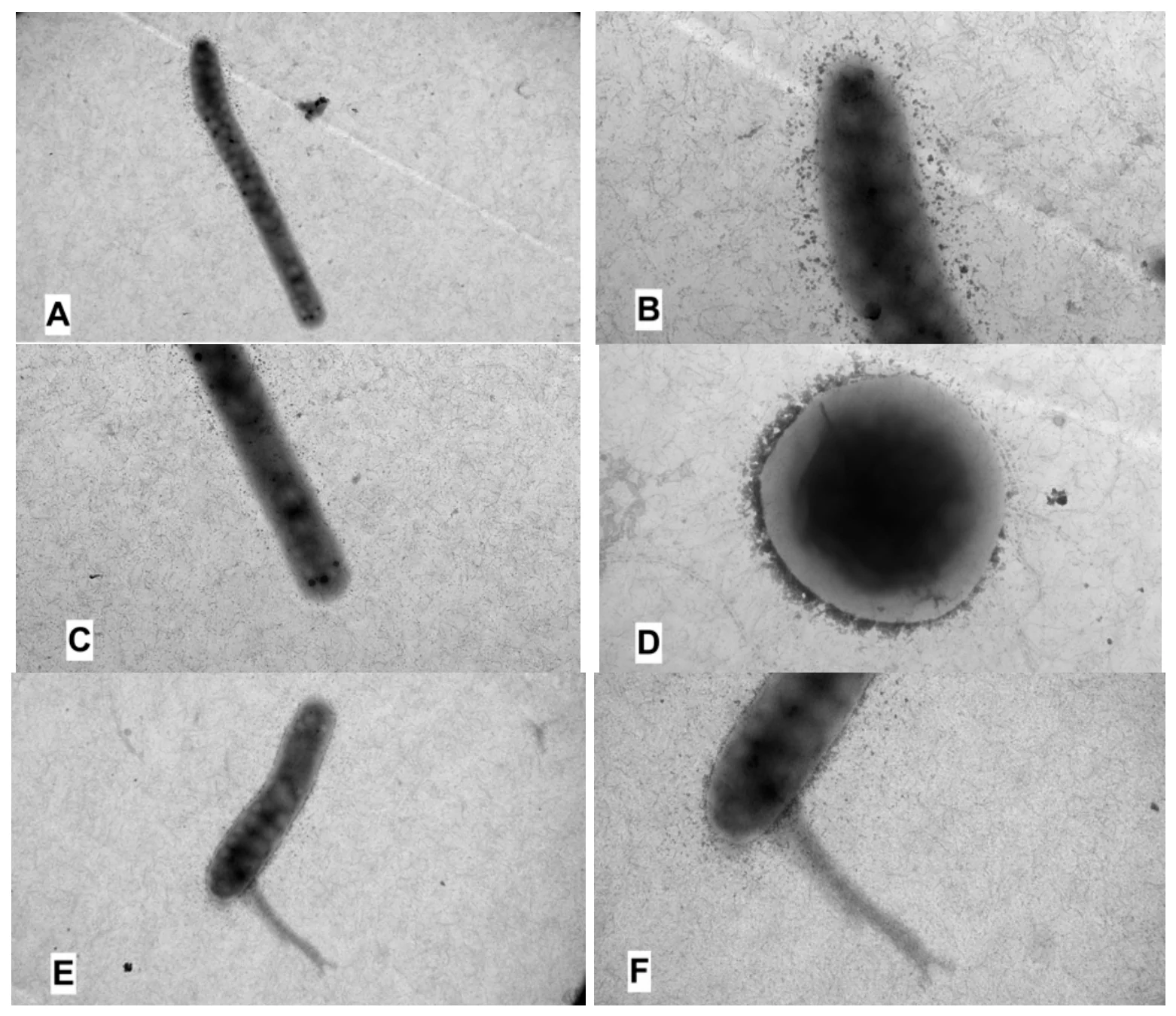
TEM images of *A. cellulolyticus* grown on MCC after a 30-day incubation period. (**A**) Slightly curved cell surrounded by particulate material, 1 µm scale. (**B**) Higher-magnification view of the upper region of the cell shown in (**A**), 400 nm scale. (**C**) Lower region of the cell shown in (**A**), 600 nm scale. (**D**) Circular electron-dense structure surrounded by particulate material, 800 nm scale. (**E**) Slightly curved cell with a flagellum and visible cell-envelope structure, 1 µm scale. (**F**) Flagellated end of the cell shown in (**E**), 500 nm scale.

Growth of *A. cellulolyticus* on MCC was slow. Cells were detected microscopically only after prolonged incubation of more than 4 weeks, and no detectable gas formation was observed after 30- or 40-day incubation periods. Methanogen cells were not observed in electron microscopy samples from MCC cultures. Figure 8 presents TEM images of *A. cellulolyticus* cells from a 30-day-old MCC culture. Cells were observed in association with surrounding particulate material. Figure 8A–C shows different regions of the same cell, whereas Figure 8E,F shows a flagellated cell with a visible cell-envelope structure. A circular electron-dense structure was also observed; see Figure 8D. No gas production was detected from cultures grown on LB as the sole carbon source.

## 4. Discussion

The anaerobes used in this study are highly fastidious, and their cultivation often requires complex protocols. Oxygen exposure can adversely affect many strict anaerobes, making cultivation dependent on specialized infrastructure and careful handling procedures. Although an anaerobic chamber greatly facilitates cultivation, such infrastructure is not available in many laboratories. The novelty of this study lies not in replacing established anaerobic techniques but in evaluating a simplified, low-infrastructure workflow for culturing fastidious anaerobes relevant to lignocellulosic biomethane research. The successful maintenance of pure cultures through repeated transfers demonstrated that the workflow was suitable for cultivating *A. cellulolyticus* and *M. barkeri*. However, no methane was detected, suggesting that a metabolically active cellulolytic–methanogenic consortium was not established under the conditions tested.

Sterilization of sealed anaerobic vessels is generally recommended only in autoclaves certified for pressurized containers because of the risk of overpressure and vessel rupture. In the present study, a small dental autoclave (GETiDY, 18 L, Zhejiang Getidy Medical Instrument Co., Ltd., Jinhua, China) was initially evaluated for sterilization of sealed vessels, and, following successful tests, a standard laboratory autoclave was used with additional safety precautions. A small number of vessels lost their seals during sterilization, likely because of internal overpressure or improper crimping, and these were discarded. These observations suggest that specialized sterilization equipment may not be essential for routine anaerobic cultivation, provided that appropriate safety procedures are followed.

Although the simplified workflow enabled routine cultivation of the selected strains, recovery from −80 °C cryopreserved stock was inconsistent and often associated with prolonged lag phases. For example, only one of eight cryovials of *M. barkeri* Fusaro yielded a viable culture after thawing. This may have resulted from brief oxygen exposure during cryovial preparation and/or residual oxygen present in the cryovial headspace, as cryopreservation was performed without an anaerobic chamber. Consequently, cryopreservation, rather than routine transfer, appeared to be the principal bottleneck for long-term maintenance of cultures under the simplified workflow. Cryopreservation may be improved by adding anoxic glycerol directly to sealed anaerobic culture vessels prior to freezing, thereby minimizing oxygen exposure. Because of the limited recovery of frozen stocks, subsequent experiments were conducted using actively maintained cultures rather than thawed cryopreserved cultures.

Beyond cultivation-related factors, substrate characteristics may also have contributed to the limited consortium activity observed in this study. The LB residue used as an industrially relevant substrate consisted of approximately 50% cellulose and 30% pseudolignin [12]. Pseudolignin is a lignin-like material formed during certain steam explosion conditions and has been reported to hinder cellulose biodegradation [14,15,16]. The complexity of this substrate may have further limited microbial activity and methane production under the conditions tested. Notably, methane production from the same LB residue has previously been demonstrated using a conventional sludge inoculum [12], suggesting that the substrate itself is amenable to anaerobic conversion. This contrast is consistent with the robustness and metabolic diversity commonly attributed to mixed sludge communities relative to the defined consortium investigated here.

*A. cellulolyticus* DSM 1870 was originally described as a cellulolytic, Gram-negative, non-spore-forming bacterium [17], but is now recognized as Gram-variable because of its multilayered cell wall [18,19]. In the present study, cells appeared as straight to slightly curved rods, occasionally bearing a flagellum, while TEM observations confirmed the presence of a multilayered cell envelope consistent with previous reports [18,19]. Electron-dense and electron-lucent regions were observed in some cells, although their biological significance remains unclear. Similar surface-associated features have previously been reported in *A. cellulolyticus* grown on cellobiose [4,20].

*A. cellulolyticus* possesses a cellulolytic enzyme system comprising endoglucanases and exoglucanases, whose expression is substrate-dependent. Endoglucanase production is induced by soluble or partially hydrolyzed substrates such as cellobiose and salicin, whereas exoglucanase activity is maximized on highly ordered crystalline substrates that require direct cell–substrate contact [4]. In the present study, TEM observations were consistent with this substrate-dependent behavior. Cells grown on MCC were closely associated with particulate material, suggesting direct interaction with the substrate and possible surface-mediated degradation, whereas such attachment was not observed in cultures grown on soluble substrates (such as cellobiose). Similar surface colonization has also been reported for other cellulolytic microorganisms, including the aerobic *Cellulomonas flavigena*, which initially grows unattached before colonizing and fragmenting MCC particles [21].

Optical density measurements were unsuitable for monitoring growth in the present study because of interference from resazurin and particulate substrates. Alternative approaches such as ATP assays, protein quantification, phosphorus-based proxies [22], qPCR, or sequencing-based methods may provide more reliable estimates of microbial growth and activity.

Table 4 summarizes previous studies involving mixed and defined anaerobic consortia. Previous studies have reported that methanogens can require up to six months of adaptation before stable growth on acetate is achieved [23], suggesting that longer acclimation periods may also be required on highly crystalline cellulose and industrial lignocellulosic residues. Consistent with the literature, successful methane production from defined consortia generally requires prolonged acclimation and carefully controlled cultivation conditions. The present study primarily focuses on simplifying the cultivation of strict anaerobes rather than optimizing methane production.

Future work should focus on optimizing consortium establishment rather than the cultivation workflow itself. Consortium establishment was inferred from cultivation observations and headspace gas composition only; molecular confirmation of the presence and persistence of both consortium members was not performed. Future studies could therefore incorporate qPCR, sequencing, or fluorescence microscopy to verify consortium establishment while simultaneously optimizing cultivation conditions. Future defined consortium experiments could be performed with extended acclimation periods, optimized inoculum ratios, and more readily metabolizable substrates, such as cellobiose, prior to transfer onto MCC or LB. The absence of methane may also have been influenced by the relatively low inoculum size (2% *v*/*v* of 4-day-old cultures), although successful methane production at comparable inoculation ratios has previously been reported using the Hungate cultivation technique [3].

Overall, the results demonstrate that cultivation of the individual anaerobes was achieved more readily than establishment of a functional consortium capable of converting MCC or LB residue into methane. The primary challenge, therefore, appears to lie not in the cultivation of the individual strains but in achieving stable consortium establishment and activity under conditions relevant to lignocellulosic biomass conversion. Future studies should incorporate molecular approaches to verify consortium establishment while investigating cultivation conditions that support methane production.

## 5. Conclusions

A simplified anaerobic cultivation workflow was successfully developed for cultivating strict anaerobes relevant to lignocellulosic biomethane research without the use of an anaerobic chamber. Pure cultures of *Acetivibrio cellulolyticus* and *Methanosarcina barkeri* were successfully established, demonstrating the feasibility of this low-infrastructure approach.

Because methane was not detected from the defined consortium using MCC and industrial LB residue as substrates, the results suggest that establishing a functional cellulolytic–methanogenic consortium was more challenging than cultivating the individual strains as pure cultures. Factors potentially limiting consortium performance included acclimation time, inoculum size, substrate complexity, and cryopreservation reliability. The proposed workflow provides a practical foundation for future studies aimed at optimizing defined anaerobic consortia for lignocellulosic biomethane production.

## Figures and Tables

**Table 1:** Gibbs free energy changes for methanogenesis using acetate, hydrogen, and methanol as substrates at neutral pH.

Substrate	Reaction	∆G° (kJ mol^−1^_methane_)
Acetate	CH_3_COO^−^ + H_2_O → CH_4_ + HCO_3_^−^	−31.0
Hydrogen	4H_2_ + CO_2_ → CH_4_ + 2H_2_O	−135.6
Methanol	CH_3_OH + H_2_ → CH_4_ + H_2_O	−112.5

**Table 2:** Headspace concentrations of methane, carbon dioxide, and hydrogen in pure cultures of *A. cellulolyticus* and *M. barkeri* strains. Values are expressed as mol % and reported as average ± standard deviation of three biological replicates; the remaining headspace gas was nitrogen.

Anaerobe	Culture Age (Day)	CH_4_	CO_2_	H_2_
*Acetivibrio cellulolyticus*				
Cellobiose	20	0.08 ± 0.01	6.85 ± 1.62	0.71 ± 0.64
MCC	21	0.01 ± 0.00	4.01 ± 1.89	0.44 ± 0.36
*Methanosarcina barkeri*				
Fusaro	20	47.73 ± 0.40	n.d.	n.d.
Schnellen	20	50.26 ± 0.46	13.56 ± 3.18	0.06 ± 0.04

n.d. not detected.

**Table 3:** Headspace concentrations of methane, carbon dioxide, and hydrogen in 4-day-old pure cultures of *A. cellulolyticus* and *M. barkeri* strains. Values are expressed as mol % and reported as *average* ± standard deviation of three biological replicates; the remaining headspace gas was nitrogen.

Anaerobe	CH_4_	CO_2_	H_2_
*Acetivibrio cellulolyticus*			
Cellobiose	0.05 ± 0.01	18.83 ± 2.79	3.85 ± 1.43
MCC	0.01 ± 0.00	2.01 ± 0.85	0.51 ± 0.38
*Methanosarcina barkeri*			
Fusaro	61.63 ± 5.34	n.d.	n.d.
Schnellen	55.41 ± 3.73	15.88 ± 4.67	1.95 ± 0.63

n.d. not detected.

**Table 4:** Summary of defined and mixed anaerobic microbial consortium studies for methane production from cellulosic substrates.

Consortium/Organisms	Substrate	Reported Anaerobic Cultivation Method	Culture Validation Method	Gas Formation	Main Findings	Notes	References
Enriched/stabilized mixed consortium (undefined)							
Mixed anaerobic consortium from municipal sewage sludge	Cellulose	Chemically defined medium; N_2_/CO_2_ (4:1 *v*/*v*) passed over a heated copper catalyst to remove traces of O_2_	Cellulose degradation, total gas, CH_4_%, pH	CH_4_ + CO_2_	Cellulose degradation: 92 to 96% (medium IV). Gas yield: ~710 mL per g cellulose degraded (with CH_4_ 51 to 56%, the rest was CO_2_) Developed a defined nutrient medium for mixed anaerobes supporting methane production from cellulose.	30 L fermenter at 35 °C repeated-fed batch culture. Sustained performance for 2 years verified stability of cellulose degradation.	[13]
Enriched anaerobic lignocellulolytic microbial consortium (rumen-derived; anaerobic fungi, bacteria, methanogens)	Cellulose (Avicel PH-101) Rice straw: cellulose (35.66 ± 0.24%), hemicellulose (18.70 ± 0.16%), and lignin (8.62 ± 0.12%)	Consecutive batch subculturing under anaerobic conditions to enrich and stabilize the consortium	VS removal, CH_4_, qPCR, ITS/16S sequencing, metagenomics	CH_4_ + CO_2_	Stable enriched consortium achieved 84% rice straw degradation and 310 mL CH_4_ g^−1^ VS added.	Demonstrates the effectiveness of long-term enrichment and acclimation of mixed consortia rather than defined cultures.	[24]
Defined consortium							
Monoculture: *Acetivibrio cellulolyticus* Co-culture: *A. cellulolyticus* + *Methanosarcina barkeri* Tri-culture: *A. cellulolyticus* + *Desulfovibrio* sp. + *M. barkeri*	CF-11 cellulose powder (Whatman) (0.25 g per 50 mL medium)	Medium preparation under 20% CO_2_–80% N_2_ using the Hungate technique	Fermentation byproducts: Ethanol, acetate, reducing sugars, CO_2_, H_2_, CH_4_	Monoculture: H_2_ + CO_2_ Co- and tri-culture: CH_4_ + CO_2_	Monoculture: Produced acetate, ethanol, H_2,_ and CO_2_ from cellulose. Co-culture: 2–3-week lag period for acetate utilization. Tri-culture: Produced the highest amount of CH_4_. Cellulose degradation: 85% of initial value, increased to 90% by increasing the population of the methanogen.	Medium inoculated with 4-days-old cultures at 2% *v*/*v* inoculum of each culture of desired anaerobe. Critical role of H_2_ in headspace to dictate fermentation outcome via interspecies electron transfer (e.g., ethanol utilization by *Desulfovibrio* only at low H_2_ partial pressure facilitated via co-culture).	[3]
Monoculture: *Acetivibrio cellulolyticus* Co-culture: *A. cellulolyticus* + *Methanosarcina barkeri*	Tissue paper (facial, 15 g per L) Cellulase assay: Carboxymethyl cellulose (CMC) or Avicel cellulose Acetate adaptation: sodium acetate	Pre-reduced synthetic salt-vitamin medium; acetate-adapted *M. barkeri*; 20% CO_2_–80% N_2_ headspace and incubation at 37 °C	Gas volume and composition (CH_4_, CO_2_, H_2_, N_2_), volatile fatty acids, cellulose degradation, cellulase activity	CH_4_ + CO_2_	Co-culture: first week of incubation, only cellulose degradation, no CH_4_ formed; 3–6 weeks of incubation, 3 g per L per week and 2.6–3.0 mol CH_4_ per mol of cellulose (glucose equivalents).	4–6 weeks of adaptation for *M. barkeri* to grow on acetate under a 20% CO_2_–80% N_2_ gas atmosphere. The co-culture exhibited an additional 2–3-week lag before efficient acetate utilization and sustained methane production.	[8]
*Ruminiclostridium cellulolyticum*, *Methanospirillum hungatei*, *Methanosaeta concilii*, *Desulfovibrio vulgaris*	Cellulose	Pure cultures revived in organism-specific media from previous protocols; methanogens acclimated in RST medium with 20% CO2–80% headspace (50 mM acetate); mixed cultures grown anaerobically in 160 mL serum bottles containing 20 mL modified VM medium with cellulose (10 g L^−1^) and 3% H_2_–97% N_2_ headspace; incubated at 34 °C for 7 days	Cellulose consumption, CH_4_, CO_2_, H_2_, H_2_S, acetate, lactate, glucose, cellobiose; metaproteomics; stoichiometric modeling	CH_4_, CO_2_, H_2_ (±H_2_S with sulfate)	Highest CH_4_ production from quad-culture attributed to positive synergy of four species, but lower cellulose degradation than the additive effects of the tri-cultures.	Focused on microbial interactions and metabolic modeling, not development of a simplified cultivation workflow. Cellulose degradation showed negative synergy, whereas methane production showed positive synergy in the four-species consortium.	[2]
Anaerobic rumen fungus (*Neocallimastix frontalis* strain PN1) + *Methanobrevibacter* sp. RA1 + *Methanosarcina barkeri* strain 227	Cellulose: Whatman No. 1 filter paper, sisal fiber, barley straw	Hungate anaerobic technique. Cultures maintained and subcultured in Hungate tubes containing 12 mL of medium with a 70% N_2_-30% CO_2_ headspace. Stable fungus–*Methanobrevibacter* co-culture established before addition of *M. barkeri*. Incubated at 38 °C without shaking	Cellulose degradation, CH_4_, CO_2_, H_2_, acetate, formate, lactate, ethanol, soluble sugars; fungal and methanogen CFU enumeration	CH_4_, CO_2_	Triculture achieved nearly complete cellulose degradation (>98%) and produced ~2 mol CH_4_ per mol hexose, outperforming the corresponding co-cultures. Methane was also produced from plant fibers (sisal and barley straw).	Stable fungus–*Methanobrevibacter* co-culture could be maintained by routine transfer, but a stable triculture could not, likely because of the slow growth of *M. barkeri*.	[25]
*Clostridium cellulovorans* + *Methanosarcina barkeri* *Clostridium cellulovorans* + *Methanosarcina mazei*	Cellulose (3 g per L)	Pure cultures cultivated using published anaerobic protocols before co-culture, incubated at 35 °C without shaking	CH_4_, H_2_, formate, acetate, RT-qPCR	CH_4_	Co-culturing with either methanogen enhanced cellulose degradation. CH_4_ yield of the *C. cellulovorans*–*M. barkeri* co-culture was 0.87 ± 0.02 mol per mol glucose equivalent.	Methanogens required ~6 months of acclimation to utilize acetate before stable growth; thereafter, cultures were routinely sub-cultured every 2 weeks.	[23]
*Acetivibrio cellulolyticus* DSM 1870 *Methanosarcina barkeri* Schnellen ATCC 43569 *Methanosarcina barkeri* Fusaro ATCC BAA-2329	Cellulose (Avicel, 5 g per L) Industrial LB residue (5 g per L)	Simplified cultivation workflow	Microscopy and gas chromatography (CH_4_, CO_2_, H_2_, N_2_)	H_2_, CO_2_, CH_4_ (not detected in defined consortium)	Simplified workflow enables pure cultures of strict anaerobes without specialized anaerobic cultivation infrastructure. Requires optimization, potentially including longer acclimation periods and higher inoculation ratio.	Cryopreservation technique requires further optimization (e.g., addition of glycerol to the active culture tube, headspace O_2_ removal, use of glass ampoules instead of standard plastic cryovials).	This study

## Data Availability

Data is contained within the article.

## Abbreviations

The following abbreviations are used in this manuscript:

LB	Lignocellulosic biomass
AD	Anaerobic digestion
MCC	Microcrystalline cellulose
SEM	Scanning electron microscopy
TEM	Transmission electron microscopy
TCD	Thermal conductivity detector
SE	Secondary electrons
BSE	Backscattered electrons
OD	Optical density

## Appendix A

### Appendix A.1

Samples were prepared using the sputtering technique (Hummer VI from Anatech, Ltd., Hayward, CA, USA) which involves depositing atoms that are torn from a metal (platinum or gold/palladium) target using ionized argon gas inside a vacuum chamber, onto the samples in a very thin layer of few nanometers to ensure that samples introduced in the SEM are conductive so as to counter the charging effects that are the cause of several imaging artifacts that often render observation impossible. The culture sample was filtered and retained on 0.2 um filters, then prepared for SEM using the following steps:

▪ Rinsing with physiological buffer before fixation if necessary.
▪ Fixation with either of the following:
  ○ 2.5% Glutaraldehyde in 0.1 M Cacodylate buffer;
  ○ 1% Paraformaldehyde + 2% Glutaraldehyde in PB buffer → 2 h.
▪ Washing.
  ○ M Cacodylate buffer or PB buffer → 2 × 5 min, or longer depending on the sample size.
▪ Post-fixation.
  ○ 2% Osmium tetroxide (OsO_4_) in 0.1 M Cacodylate buffer or PB buffer → Time: 1 h.
▪ Washing with either of the following:
  ○ M Cacodylate buffer or PB buffer → 2 × 5 min, or longer depending on the sample size.
  ○ Pure (bi-distilled) water → 2 × 5 min, or longer depending on the sample size.
▪ Storage in pure water overnight (or for a few days, but no more).
▪ Dehydration (each step done for 5–15 min):
  ○ Ethanol 50%.
  ○ Ethanol 70%.
  ○ Ethanol 85%.
  ○ Ethanol 95%.
  ○ Ethanol 100% (×3).
▪ Critical point drying (CPD) using CO_2._
  ○ Let the dried specimens cool down in a dry bell jar (with drierite desiccant).
▪ Mounting on appropriate supports (stubs).
  ○ Use carbon adhesive tabs, double-sided tape, or silver paint directly.
  ○ Use silver paint to ensure conductivity between the sample and the stub.
▪ Metal coating.
  ○ Generally done with gold–palladium (Au-Pd) (mentioned above).

Transmission electron microscope (TEM) protocol:

▪ Fix for a minimum of 4 h at 4 °C with 2.5% glutaraldehyde in 0.1 M cacodylate at pH 7.2.
▪ Wash 3× using cacodylate buffer and centrifuge at 2000× *g* 5 min.
▪ Remove supernatant and immobilize with the same amount of low-melting-point agarose (3% final).
▪ Cut into strips of 0.5 by 0.5 by 5 mm and wash 2× in cacodylate buffer.
▪ Post-fix in 1% osmium tetroxide (OsO_4_) for 1.5 h at room temperature.
▪ Wash 3× in cacodylate buffer.
▪ Dehydrate with grading ethanol (30%, 50%, 70%, 95%, 100%, 5 min each).
▪ Wash 3× 5 min with 100% ethanol.
▪ Transfer and wash 2× in 100% propylene oxide.
▪ Transfer to epon-8121 (without hardener) 50% in propylene oxide and incubate 16 h in a rotary mixer.
▪ Remove caps and evaporate propylene oxide.
▪ Replace with epon-812 and incubate for 16 h in a rotary mixer.
▪ Transfer to freshly mixed epon and hardener (BDMA or DMP-30) and incubate for 2.5 h.
▪ Include in silicone mold and incubate 3 days at 60 °C.
▪ Cut sections with a Leica ultramicrotome using a 35° diatome diamond blade.
▪ Sections are stained for 10 min in 2% uranyl acetate and 5–10 min in 3% lead citrate.

### Appendix A.2

The gas chromatography method was verified using a gas standard prepared using a gas mixing bench. The measured gas compositions agreed closely with the prepared standard (Table A1).

**Table A1:** Calibration range for each component measured by the gas chromatography method, and verification using a prepared gas standard.

Gas Component	Calibration Range (mol %)	Prepared Gas Standard (mol %)	Measured by GC (mol %)
H_2_	1.09–70.47	20.4 ± 0.1	21.5 ± 0.8
CO_2_	1.20–70.00	20.6 ± 0.2	20.1 ± 0.6
CH_4_	2.29–94.85	60.1 ± 0.1	59.7 ± 1.1
